# THE Impact of Disruption on the Relationship Between Exploitation, Exploration, and Organizational Adaptation

**DOI:** 10.3389/fsoc.2021.757160

**Published:** 2021-11-03

**Authors:** Yao Xiao, Jie Cen, Peder Soberg

**Affiliations:** ^1^ School of Business Administration, Zhejiang Gongshang University, Hangzhou, China; ^2^ DTU Engineering Technology,Technical University of Denmark, Ballerup, Denmark

**Keywords:** disruption, exploration, exploitation, organizational adaptation, organization learning

## Abstract

Firms should deploy exploration and exploitation to foster organizational adaptation. Previous research on exploration and exploitation lacked a focus on disruption implications in different contexts. This study aims to empirically test a moderation model including disruption events, exploration, exploitation, and organizational adaptation and enable a deeper understanding of organizational learning and innovation theory to yield competitive advantage and sustainability of innovative firms. Our results reveal that exploration is more effective during outside disruption events. The results do not support the concept that exploitation is more effective during inside disruptions. Disruptions also moderate the combined effect of exploration and exploitation. Although they are generally complementary in facilitating organizational adaptation, a singular focus on either exploration or exploitation is as effective as is combining exploration and exploitation during inside and outside disruption events. The results of an event study using seven Chinese international firms, including Alibaba, Meituan, Dianping, Baidu, Beibei, TP-link, and Maxio, provided 132 completed and usable questionnaires that supported our hypotheses. Our study contributes to a better understanding of disruption, exploration, exploitation, and related performance implications.

## Introduction

Firms need to continuously exploit and explore to remain competitive in both the short and long term (March 1991). Most organizational learning studies present exploiting knowledge from within the organization or exploring knowledge from outside the organization as two learning methods that enhance performance (Boumgarden et al., 2012).

Few studies on the relationship between exploration, exploitation, and organizational adaptation, focus on the influence of the context, including whether it is disruptive. Furthermore, although studies on organizational learning generally show that learning from experience benefits organizational performance over time (Argote, 1999), other research suggests that knowledge accumulated from experience sometimes creates rigidities that disrupt learning and harm performance (Leonard-Barton, 1992). These and other examinations of organizational learning tend to focus on disruptive innovation and technology. Furthermore, relatively few studies examine how organizational learning affects organizational adaptation in a specific context with or without disruption (Piao and Zajac, 2016; Paruchuri and Awate, 2017).

Past studies emphasize the role of disruption in exploration and exploitation (Barnett and Freeman, 2001) but essentially neglect disruptive events at the organizational level (Ren et al., 2019). Few studies analyze organizational learning and adaptation under diverse disruptive circumstances (Døjbak Håkonsson et al., 2016). Although the significance of these studies is undeniable, we need to further our understanding of how to motivate organization members’ exploration and exploitation in ways that lead to organizational adaptation in a disruptive context (Engman, 2019).

This paper analyses how disruption influences the relationship between exploration, exploitation, and organizational adaptation. Mainly, we aim to explain and resolve the conflicting empirical evidence in the existing literature on differences in the efficacy of exploration and exploitation. Using data from the Chinese Internet enterprises Alibaba, Meituan, Dianping, Baidu, Beibei, TP-Link, and Maxio, we examine various factors and events that likely facilitate or destruct organizational adaptation. Second, we aim to explain the moderation effect of various disruption events outside or inside the organization. Finally, by examining the effects of these disruption events on organizational adaptation, our research clarifies the best approach for organizational adaptation.

This paper contributes to the literature on exploration and exploitation (Aggarwal et al., 2011; Boumgarden et al., 2012; Mudambi and Swift, 2014; Choi and McNamara, 2018) with insights into how disruption events shape an organization’s exploration and exploitation learning. This paper provides evidence of how organizations explore, exploit, and switch between both (Gambeta et al., 2019; Aznar-Sánchez et al., 2019). This paper also contributes to the literature on disruptive innovation with two types of events by providing further insights into the heterogeneous effects of exploration and exploitation on organizational adaptation.

## Theoretical Development

### Organizational Adaptation

Organizational adaptation is important for firm survival, for competitive strength (Ali Hameed et al., 2019; Ali Hameed et al., 2019). Organizational adaptation represents the critical ability to fit the environment or the ecosystem to survive and grow (Bode et al., 2011). Organizations adapt over time, research suggests that organizational adaptation to opportunities arising in the external environment as precondition for organizational growth and survival (Schmitt, 2015). Parallel with the above mentioned research stream (Bansal, 2014) believe the ability to respond and adapt to environmental changes has become a vital success factor for organization. In many industries, firm intiate response actions reactively or proactively to overcome poor adaptability and avoid performance deterioration (Slawinski, 2012). Despite these emerging research insights, several scholars have recently emphasized the relationship between organization learning and organizational adaptation (Hoever et al., 2018; Kaiser et al., 2018; Kim et al., 2019). This scholars highlight the importance of difference learning method when analyzing organizational adaptation to rate of internal and external changes. Building on this insight, Posen et al. (2018) highlights collaboration between organization members or departments play a vital role to improve organizational adaptation. In a similar vein, Ethiraj and Levinthal (2014) emphasizes the capability of independent innovation as a dimision of organizational adaptation. Therefore, we define organizational adaptation as a critical ability to fit environment or ecosystem to survive and grow, which consisted with knowledge learning, collaboration and capability of independent innovation.

### Exploitation and Exploration

The two learning methods, exploration and exploitation, enable different types of organizational adaptation (Angeler et al., 2019) and performance implications (Posen et al., 2018). However, exploration and exploitation learning do not always increase the performance or adaptation of an individual organization (Yang et al., 2015). Exploration and exploitation respectively create different results under disparate circumstances (such as employee turnover, technology change, business model change, and others) (Swift, 2016). Organizational knowledge is dynamic and encompasses codified, procedural knowledge embodied in organizational goals, routines, standard operating procedures, and rules through the learning loop (Hongyan Yang et al., 2010; Yu et al., 2019). The literature on organizational learning suggested that individuals exploit existing knowledge rather than explore new knowledge because of the high chance of exploration failure (Stettner and Lavie, 2014; Kim and Anand, 2018). Organizations learn more effectively from failure than from success (Madsen and Desai, 2010). Organizational failure brings experience for the collective and is an opportunity that enables individuals to figure out the scarce resource or ability that they neglect (Posen et al., 2018). However, why do exploration and exploitation learning have disparate influences on organizational adaptation? We can think of several significant reasons.

One answer is that exploration and exploitation learning have different connections between tacit and explicit knowledge (Paruchuri et al., 2006). Exploration learning is better for explicit knowledge because this type of knowledge is easier to discover and utilize when organizations need to create new knowledge (Jain, 2016; Chen et al., 2020). In contrast, tacit knowledge improves performance when the environment demands high levels of exploitation behavior (Gambeta et al., 2019; Wang et al., 2019). Moreover, exploration and exploitation have disparate influences related to weak and strong ties. Strong embedded ties help firms increase performance (Aranda et al., 2017; Choi and McNamara, 2018; Zhou and van Knippenberg, 2018). However, over-embeddedness can hurt economic performance by making firms vulnerable to environmental change attributable to the limited diversity of information to which they have access (Paruchuri and Awate, 2017). Additionally, strong ties are better for performance when the environment demands high levels of exploitation behavior, but weak ties improve performance when the environment demands high levels of exploration behavior (Moreira et al., 2018).

In different types of organizational ecosystems and environments, exploration and exploitation create different performance effects (Yang et al., 2014). Lee and Meyer-Doyle (2017) examined how incentives influence whether individuals explore new ideas or exploit existing ideas and found that individuals engage in relatively more exploration under weakened performance-based incentives. Desai (2015) tested related hypotheses on a hospital panel and found that organizations learn less effectively when their failures are concentrated in origin, involving a particular unit or individual. Learning from experience benefits organizational performance over time (see reviews by Argote (1999)). However, knowledge accumulated from experience can sometimes create rigidities that disrupt learning and harm performance (e.g., Leonard-Barton, 1992; Levitt and March 1988; March 1991; Tushman and Romanelli, 1985). These studies suggested that, on the one hand, different types of organizational ecosystems and environments (e.g., employee turnover, technology change, or extreme events) are shift parameters for exploration learning and exploitation. However, we need to better understand when exploitation and exploration occur and how they impact (Paruchuri and Awate, 2017; Moreira et al., 2018). On the other hand, the negative factors surrounding the organization’s learning are not always harmful to organizational performance.

In summary, the relationship between exploration learning, exploitation learning, and organizational adaptation is quite different when the environment changes or under disparate circumstances (Uzunca, 2018). Recent literature realized the importance of exploration and exploitation for adaptation and performance (Cui et al., 2018). This study combines disruptive innovation theory and organizational learning theory to address the research gap.

### Disruption Events

Joseph Schumpeter (1939) proposed the disruption innovation theory, including *disruption technology* and *disruption innovation*. Disruption technology will cause mutations in existing market structures and cheaper, simpler, and more convenient technologies. Christensen and Bower. (1996) proposed two types of disruptive innovation theory: low-end disruptive innovation and new market disruptive innovation. He pointed out that low-end disruptive innovation such as organization task change more likely occurs internally in the organization. Market disruption innovation likely takes place externally to the organization. Anderson and Lewis (2014) define disruptive organizational events as technological change and restructuring and extreme events, such as disruptive technology and disruptive innovation. They point to the positive impact of disruptive events on organizational learning.

Existing research has found two completely different phases of disruption (Donald and William, 2016): disruption in the first phase is mainly within the enterprise, such as the organizational tasks change. Disruption in phase two is more widespread and exists in the market or industry outside of the organization. Much research focuses on this type of disruption (Carnabuci, 2010). Hale et al. (2016) divides disruption events into two stages: the impact on collective performance and the recovery cycle. Lange et al. (2009) found that disruption events promote organizational innovation.

### Impact of Inside Disruption Events

We identify two distinct types of disruption events closely related to the learning curve and organizational change theory. Specifically, we conceptualize employee turnover and restructuring as inside disruption events that stem from within the organization (Anderson and Lewis, 2014). Employee turnover generally hurts organizational adaptation not only because the organization loses accumulated individual knowledge when employees leave but also because employee turnover disrupts existing routines for interacting and accomplishing tasks (Kilkki et al., 2018; Mevada et al., 2019). Employee turnover or restructuring harms existing strong ties and tacit knowledge^1^ (Granovetter, 1973). However, employee turnover or restructuring may benefit explicit and collective knowledge long-term (Piao and Zajac, 2016; Sørensen, 2019). In particular, this social network view of inside disruption events implies that employee turnover or restructuring influences organizational resources and adaptation (Mata and Alves, 2018). Moreover, indirect evidence from other studies suggested that the positive effects of employee turnover might be attributed more to exploitation than exploration (Sawik, 2019).

Employee turnover and restructuring likely disrupt individual knowledge but foster collective knowledge (Rhee and Leonardi, 2018). Furthermore, employee turnover disrupts organizational routines but creates new ones and increases explicit knowledge, beneficial for organizational adaptation in high exploitation environments (Paruchuri and Awate, 2017). Disruption events, such as individual turnover, may benefit organizational performance through the long-term disruption of the accumulated experience (Hale et al., 2016). Inside disruption events can positively moderate the effect of exploration and exploitation learning on organizational adaptation but more so for exploitation (Bode et al., 2011; Cohendet and Simon, 2016). Combining the theory of organizational learning and organizational change shows that employee turnover does not destroy the knowledge store at the collective level. Accordingly, an organization’s members prefer using existing knowledge and resources to deal with the crisis instead of exploring new resources because exploration always comes with huge costs. Employee turnover transforms knowledge rather than damages it (Piao and Zajac 2016; Schilling and Fang, 2014). Therefore, under disruption events from within the firm (e.g., employee turnover and restructuring), exploration learning increases organizational adaptation more than exploitation learning. Finally, we believe that the positive influence of exploitation learning on organizational adaptation is stronger in inside disruption events than outside disruptions.

H1: During inside disruption events, exploitation has a stronger positive effect on organizational adaptation than exploration.

### Impact of Outside Disruption Events

We conceptualize outside disruption events as changes in technology and extreme events that stem from outside the organization. As we know, changes to tasks or innovations in technology can render prior knowledge less valuable or even obsolete (e.g., Polidoro, 2013; Tripsas, 1997; Tushman and Anderson, 1986). For example, individuals’ knowledge can become useless when facing situations where the old knowledge is no longer relevant, or the task requires new learning (chase and Simon 1973). Anderson and Lewis (2014) used a simulation method to test the influence of environmental disruptions on individual and collective knowledge. They emphasized that technology change or a firm’s extreme events (unforeseen business model or environment changes) disrupt both individual and collective knowledge short term but increase collective knowledge long term. Explicit and tacit knowledge is less valuable or obsolete during outside disruptions because strong ties and trust between core employees become dysfunctional or even non-existing (Stettner and Lavie, 2014). However, these disruption events might trigger individual exploration and new weak ties with potential collaborators and may eventually increase organizational adaptation (Stieglitz et al., 2016; Swift, 2016).

Therefore, we argue that outside disruption events amplify the positive effect of exploration on organizational adaptation. An organization’s routines and the trust among old collaborators hardly solve new problems during outside disruption events because existing routines and collaboration always mean old knowledge and relationships, and firms attempt to develop new diversity resources regardless of whether at the individual or the organizational level (Haans, 2019). Second, firms prefer exploration learning in highly disruptive environments (Gambeta et al., 2019). As mentioned, individuals’ knowledge can become useless when facing a new task that requires new learning (Jarzabkowski and Lê, 2012). Individuals and the collective in organizations engage in more exploration to manage dynamic technology because individuals hardly find available existing resources to advance their ability when the environment is turbulent (Yang et al., 2015). Third, prior literature suggested that exploration and having a network with many weak ties positively affect performance in an unstable environment (Ioannou, 2014).

Firms choose different strategies and perform differently during inside and outside disruption events (Park et al., 2019). The knowledge in a firm shapes its learning methods, that is, exploration and/or exploitation learning during inside and outside disruption events foster organizational adaptation. Hence, we develop the following hypothesis.

H2: During outside disruption events, exploration has a stronger positive effect on organizational adaptation than exploitation.

### Disruption as a Shift Parameter

As previously noted, organizational adaptation represents the critical ability to fit the environment or the ecosystem to survive and grow (Bode et al., 2011). Despite the importance of organizational adaptation, organizations should avoid disruption in the long term (Gimeno and Woo, 1996). Learning from experience benefits productivity, but knowledge accumulated from experience can decrease new knowledge adoption and harm organizational performance (Piao and Zajac, 2016).

Above all, we argue that exploration and exploitation are the core resources that foster organizational adaptation, and disruption as a shift parameter moderates the relationship between exploration and exploitation learning with organizational adaptation for several reasons (Engman, 2019). First, many studies examined the role of disruptions in organizational functioning and survival. For example, Anderson and Lewis (2014) used the system dynamics methodology to model the effects of disruptive events on individual and collective learning and productivity in organizations. Learning from failure experiences or others’ failures might benefit firm success (Amburgey et al., 1993; Desai, 2015; Dowell and Muthulingam, 2017; Engman, 2019). These and other studies suggested that accumulated experiential knowledge can reduce a firm’s ability to adapt to change.

Moreover, exploration and exploitation learning influence productivity differently in disparate contexts (Ali Hameed et al., 2019). For example, Rao and Argote (2006) found that disruption events, such as employee turnover among members of groups performing a production-type task, were less harmful to overall productivity when groups were highly structured and explored. These findings are consistent with prior research that showed positive outcomes when combining exploration and exploitation during disruption events (Piao and Zajac, 2016). Unfavorable circumstances influencing organizational adaptation include employee turnover or technology change (Anderson and Lewis, 2014). Organizational change and innovation theory conceptualize disruption in several ways. Some disruption events stem from changes inside the firm, such as employee turnover and restructuring, and some disruptions events stem from changes outside, such as technological change and extreme events (Bode et al., 2011). Each of these events will affect the relationship between exploration, exploitation, and organizational adaptation.

**FIGURE 1:**
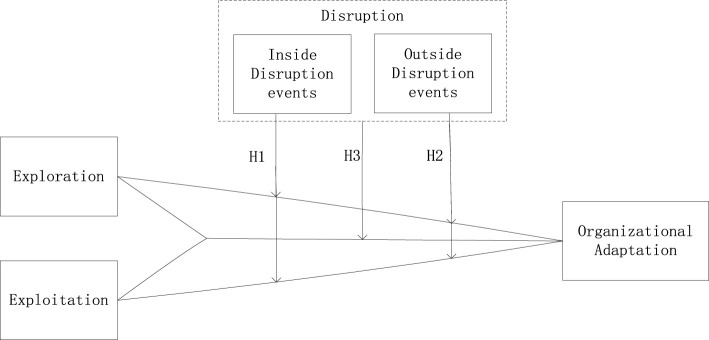
The conceptual model.

In summary, exploration and exploitation learning affect organizational adaptation. We use the learning curve and organizational change theory to argue that disruption events moderate their relationship. Hence, we develop the following hypothesis. The conceptual model as showed in Figure 1.

H3: Disruption events moderate the effect of exploration and exploitation on organizational adaptation.

## Methodology

### Data Collection

We tested our hypotheses using a questionnaire survey of seven Internet firms, which resulted in 132 questionnaires from Alibaba, Baidu, Meituan, Dianping, Beibei, TP-link, and Maxio. There are several reasons for selecting this sample. First, the industry competes in a dynamic technological environment coping with rapidly external context and disruption. Second, the internet industry put massive R&D inputs on innovation, increasing their organizational adaptation. The questionnaire survey consists of two phases. First, we invited ten Internet firm engineers for individual interviews. Using questionnaires, we interviewed them about organizational learning during disruption events, such as employee turnover, restructuring, technology change, and extreme events. The interviews verified that companies utilize different learning methods during various disruption events to improve productivity and organizational performance and adapt to the changes and innovation of the external environment. The interviews initiated the questionnaire distribution.

The 10 Internet engineers work at Alibaba, Baidu, Meituan, Dianping, Beibei, TP-link, and Maxio. Alibaba, Baidu, Dianping, and Beibei are software Internet firms. Meituan, TP-link, and Maxio are hardware Internet firms. The interviewees engaged in snowballing and semi-structured interviews; through them, we sent 200 questionnaires to other connections at these firms. The approach resulted in 157 returned questionnaires, and 132 were valid. Table 1 indicates a questionnaire recovery of 66% and the sample characteristics distribution of the questionnaire.

**TABLE 1:** The sample characteristics distribution of the questionnaire.

	Item	Number	Ratio (%)		Item	Number	Ratio (%)
Co.NAME	Alibaba	34	25.77	GENDER	F	86	65.15
	Meituan	21	15.92		M	46	34.85
	Dianping	15	11.36	POSITION	Top manager	15	11.37
	Baidu	16	12.12		Middle manager	23	17.42
	Beibei	7	5.31		Technical staff	94	71.21
	TP-Link	19	14.35	WORKING	1–3	80	60.61
	Maxio	20	15.17	EXPERIENCE	3–6	36	27.27
Co.SCALE	Below100	0	0.00		Beyond 6	16	12.12
	100–1,000	79	59.85	AGE	Below29	56	42.42
	1,000–10,000	19	14.40		30–39	58	43.94
	Beyond10000	34	25.75		40–49	18	13.64

### Dependent Variable

**Organizational adaptation (OA).** Organizations adapt over time, but both selection and adaptation drive change (Levinthal, 1991). We followed Ethiraj and Levinthal (2014) to measure the firm-level OA as a dependent variable. They believed that innovating and updating existing knowledge constitute OA elements. Therefore, our data on OA consist of five items: dynamic adaptability to the environment, ego organizational learning, updating and reviewing existing knowledge, having the capability of independent innovation, and the organization’s flexible collaboration (Posen et al., 2018; Ali Hameed, 2019).

### Independent Variable

**Exploration and exploitation (ER and ET).** We use the scale of exploration and exploitation learning from March (1991), which proved to have high reliability and on which other studies were also developed (e.g., Boumgarden et al., 2012; Døjbak Håkonsson et al., 2016; Jiang et al., 2017; Paruchuri and Awate, 2017; Choi and McNamara, 2018; Dattée et al., 2018). To collect data for these measures, we asked Internet firm members to indicate using a 1–5 Likert scale the extent to which six different statements regarding the exploration and exploitation ability of their firms were true. Three of the statements concerned the firm’s exploration (i.e., top manager encourages innovation, leaders’ decision making, new technology ability, rewards for technology innovation). Three items pertained to exploiting (flow of new knowledge, balancing new and original technology, and using acquired new knowledge and technology). The Cronbach’s alphas for the exploration measure are 0.65 and 0.73 for exploitation.

**Inside and outside disruption (ID and OD).** We use the scale of ID and OD from Anderson and Lewis (2014), Engman (2019), and Dattée, Alexy, and Autio (2018), which have high reliability. Other studies used these scales (e.g., Goodman and Leyden, 1991; Lewis et al., 2007; Pisano et al., 2001; Mevada et al., 2019; Vega et al., 2019). To collect data for these measures, we asked Internet firm members to indicate using 1–5 Likert scales the extent to which six different statements were true regarding disruptive events at their firms. The variable for disruption inside a firm includes three items: employee turnover, restructuring, and task change (Anderson and Lewis, 2014). Disruption from outside a firm consists of three items: market structure change, technology change, and extreme events (Anderson and Lewis, 2014; Engman, 2019). The Cronbach’s alphas for the ID measure are 0.77 and 0.86 for the outside disruption measure.

We employed confirmatory factor analysis to examine the validity of the OA, exploration, exploitation, and disruption scales. The fit indices showed that the measurement model fit the data reasonably well ( ${\_}^{2}$ = 69.393, *p* < 0.05; comparative fit index = 0.845; normed fit index = 0.735; root mean square error of approximation = 0.079; standardized root mean square residual = 0.054), and all of the items in these three constructs have highly significant standardized loadings. Using these loadings, we found the composite reliability for organizational adaptation (0.66), exploration (0.65), exploitation (0.73), ID (0.71), and outside disruption (0.86). Thus, these measures demonstrate good convergent validity and reliability.

**Control variables.** In our analyses for H1, H2, and H3, we control for the following: firm age and firm size (FA and FS), knowledge diversity (KD), accumulated experience diversity (AED), technological dynamism (TD). Prior studies identify these variables as factors that can affect the overall exploration, exploitation (Yang et al., 2015; Yan and Chang, 2018), and organizational adaptation during a disruption. Table 2 shows each index measures item in detail.

**TABLE 2:** Each index measures item in detail.

First-class indexes	Second-class indexes	Items		References
DISRUOTION	Inside	D1	Employee turnover	Anderson and Lewis (2014)
	disruption events	D2	Restructuring	Fisher and White (2000); chase &Simon (1973)
		D3	Task change	Jarzabkowski and Lê. (2012)
	Outside	S1	Market structure change	Mevada et al. (2019); Vega et al., (2019)
	disruption events	S2	Technology change	Engman (2019)
		S3	Extreme events	Anderson and Lewis (2014)
DUAL	Exploration	E1	Top manager encourage innovation	March (1991); Jiang et al. (2017)
LEARNING	learning	E2	Leaders’ Decision-making ability to new technology	Dattée et al. (2018)
		E3	Rewards for technology innovation	Choi and McNamara (2018)
	Exploitation	L1	The flow of new knowledge	Mudambi and Swift (2014); Paruchuri and Awate (2017)
	learning	L2	Balance of new and original technology	Stettner and Lavie (2014); Yang et al., 2015
		L3	Using acquired new knowledge and technology	Moreira et al. (2018); Døjbak, Håkonsson et al. (2016)
ORGANIZATIONAL		A1	Dynamic adaptability of the environment	Levinthal (1991)
ADAPTATION		A2	Ego organization learning	Levinthal and March (1993)
		A3	Update and review exist knowledge	Ali Hameed et al. (2019)
		A4	The capability of independent innovation	Ethiraj and Levinthal (2014)
		A5	Flexible collaboration of the department	Posen et al. (2018)

### Model

Following prior research (Levinthal, 1991), we tested our hypotheses through SPSS 25.0 by multiple regression of organizational adaptation (OA) on the key predictor variables, as represented by the following equation:

$${\text{OA}}_{\text{i}}={\text{a}}_{0}+{\text{a}}_{1}\_\text{X}+{\text{a}}_{2}\_\text{Controls}+{\text{e}}_{\text{i}}$$

$${\text{OA}}_{\text{j}}={\text{b}}_{0}+{\text{b}}_{1}\_\text{X}\_\text{Z}+{\text{b}}_{2}\_\text{Controls}+{\text{e}}_{\text{j}}$$

We use two stages to measure the independent variable and moderation effects on OA. In the first regression (see formula 1), we modeled the linear relationship among exploration, exploitation, and OA. In the second stage regression (see formula 2), we modeled the likelihood of organizational adaptation among disruption events and two learning methods (exploration and exploitation).

## Results

Table 3 reports descriptive statistics, such as mean values, standard deviations, and bivariate correlations, as evidence for the basic relationship between the independent and the dependent variables. The exploration and exploitation mean values are 2.158 and 2.635. Exploitation has a significant negative effect on outside disruption events (β = −0.054, *p* < 0.05). Between exploration and exploitation, ID and OD show no significant effect (β = −0.347, *p* > 0.05, β = −0.263, *p* > 0.05). The variance inflation factor (VIF) coefficient of each direct variable ranged from 2.147 to 4.598, lower than the critical VIF value of 10. The tolerance value ranged from 0.254 to 0.491, higher than the recommended lower limit of 0.100. The results also show that no multicollinearity problem exists in the data related to each variable.

**TABLE 3:** The means, standard deviations, and Pearson coefficients of the variables.

	Mean	Std.Dev	1	2	3	4	5	6	7	8	9	10
1.FA	4.215	5.103	1									
2.FS	1.368	0.211	0.314	1								
3.KD	17.235	10.694	0.216*	−0.315*	1							
4.AED	1.233	0.337	0.120*	0.102	0.210*	1						
5.TD	5.369	5.079	0.241**	0.301	0.090	-0.209**	1					
6.ER	2.158	0.521	0.350	0.215*	-0.315	0.327	0.217*	1				
7.ET	2.635	0.631	0.300	0.366	0.257*	0.187*	0.109*	0.347	1			
8.ID	3.254	0.577	0.039	−0.214*	0.314*	0.318	0.216	−0.125**	0.245*	1		
9.OD	3.657	0.633	0.184*	−0.169*	0.119*	0.294	0.147	0.426**	−0.054*	0.263	1	
10.OA	3.652	0.724	0.214*	−0.198**	0.264	0.158**	0.169	0.239**	0.211*	0.324**	0.366*	1

Note:*p < 0.05.**p < 0.01.***p < 0.001.

Table 4 displays the result of the regression analysis. Model one presents the baseline model only with control variables. Firm size does not moderate OA. The other control variables (firm age, KD, AED, and TD) positively affect OA. Next, we entered our main predictor variables in Model 2. After that, we added the interaction terms in Models 3–5. Model 2 exhibits a positive effect of exploration (ER) and exploitation (ET) on OA (β = 0.060, *p* < 0.05; β = 0.152, *p* < 0.05). This positive effect is significant in all models. This result confirms the relationship between exploration, exploitation, and OA found in the literature (Laureiro‐Martínez et al., 2015). Models 3 and 5 show that disruption events (DE), including inside disruption (ID) and outside disruption (OD), moderate the effects of ER and ET on OA.

**TABLE 4:** The regression analysis results.

Variable	OA							Collinearity diagnostics	
Control variables	M1	M2	M3			M4	M5	Tolerance	VIF
Firm age	0.217*	0.209*	0.245*	0.236*	0.179*	0.163*	0.222*	0.324	1.406
Firm size	0.194	0.233	0.159	0.234	0.128	0.166	0.147	0.422	1.254
Knowledge diversity	0.125*	0.214*	0.112*	0.186*	0.126*	0.254*	0.332*	0.396	2.265
Accumulated experience diversity	0.248*	0.169*	0.174*	0.135*	0.163*	0.222*	0.159*	0.347	2.954
Technological dynamism	0.158*	0.218*	0.209*	0.189*	0.166*	0.213*	0.215*	0.303	2.857
Independent variable									
ER		0.265*	0.186*	0.196*	0.224*	0.167*	0.198*	0.409	1.339
ET		0.157**	0.208*	0.245**	0.263*	0.211**	0.274**	0.398	2.014
ID		−0.358*	0.235	0.332	0.099	0.015*	0.123*	0.354	2.114
OD		−0.155	0.177	−0.125	−0.332	−0.012*	0.059	0.344	2.458
ID*EA			0.211**		0.158*			0.378	3.625
ID*EI				0.249**	0.187*			0.336	3.447
OD*EA						0.159**		0.408	3.147
OD*EI						−0.142**		0.421	3.655
DE*EAL*EI							0.287**	0.399	4.005
Observations	132	132	132	132	132	132	132		
*R* ^2^	0.048	0.189	0.254	0.249	0.297	0.306	0.358		
Adjusted R	0.046	0.185	0.253	0.244	0.296	0.305	0.357		
F	4.176*	4.366**	3.677**	3.417*	4.012*	4.219*	3.992**		

To test hypothesis 1, model 3 shows that ER and ET have a positive and significant interaction effect of ID on OA. In Model 3-1, ER exhibits a significantly positive interaction effect of ID on OA (β = 0.211,*p* < 0.01), Model 3-2, ET shows significantly positive interaction effect of ID on OA (β = 0.249,*p* < 0.01). To further test hypothesis 1, distinguishing the significant between ER and ET, the interaction effect of ID on OA, we add ER and ET in Model 3–3. The evidence shows that it is both positive and significant. Similar to Liu (2009), comparing the ⊿*R* ^2^ between different independent variables to show which one explains more the dependent variable, M3-3-M3-1 was used to calculate, ⊿*R* ^2^ where ⊿R^2^_M4–3-M4-1_=R^2^_M4–3_-R^2^_M4-2_=0.297‐0.49=0.048, which concerns moderation from inside disruption. The first formula represents the variance proportion where OA was affected by ER, and the second formula represents the variance proportion where OA was affected by ET. ⊿R^2^_M4–3-M4-1_<⊿R^2^_M4–3-M4-2_ means that ER explains more OA improvement than ET under the moderating effect of ID. The findings do not support hypothesis 1.

To test hypothesis 2, Model 4 finds that ER has a positive and significant interaction effect on outside disruption (OD) (β = 0.159,*p* < 0.01) on OA. To our surprise, on the contrary, ET has a negative and significant interaction effect on OD (β = −0.142,*p* < 0.01). This finding supports hypothesis 2, which posits that exploration has a stronger positive effect on organizational adaptation than exploitation during outside disruption events.

To test hypothesis 3, model 5, we find two learning methods (including ER and ET) together have a positive and significant interaction effect with disruption events (including inside disruption and outside disruption) on OA (β = 0.287,*p* < 0.01. The findings support hypothesis 3.

**FIGURE 2:**
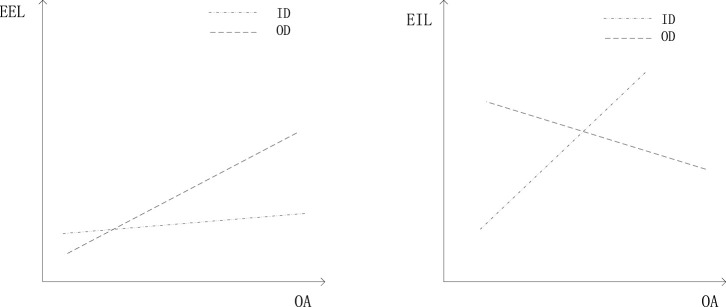
Schematic diagram for the regulating effect of variable.

To gain additional insights into these results, we graphed the interaction plot in Panel A of Figure 2. Based on our regression results, ER benefits OA during both inside and outside disruption but more so during outside disruption. Panel B of Figure 2 shows that ET benefit OA during inside disruption and harm OA during outside disruption. Also, tolerance of all variables was beyond 0.1, inflation factors (VIF) test found that all variables VIF are far below the critical value of 10, thus mitigating multicollinearity concerns.

### Post-Hoc Analyses

We conducted sensitivity tests as follows. First, we separated our primary dependent variable—organizational adaptation—into two levels: individual and collective adaptation. This change did not alter the overall pattern of our results, and our key independent variables ER and ET remained positive with significant interaction effects with ID and OD. Second, we experimented with control variables of technological capability and financing capability to account for organizational innovation ability beyond the KD and TD. The rationale is that technology and financing capabilities are more explicit than TD. This additional control showed a non-significant impact on the dependent variable and made little difference to our key findings (the findings support hypothesis 2). Lastly, we explored innovation performance as our dependent variable because innovation performance is a key factor for OA. The findings indicate that ER and ET have an independent positive effect on organizational innovation performance, including interaction effects with DE. This pattern of results suggests that our theory is also applicable to organizational innovation, although weaker than with OA. The appendix includes the robustness results.

## Simulation Experiments

In order to further verify the results of the empirical study in this paper, we run a computer simulation with MATLAB 2016a software. We analyze the different stages and different levels (e.g., individual level and collective level) concerning innovative organization, organizational learning, and knowledge acquisition. Specifically:

### Variable Setting

*Disruption events.* Organizational disruption negatively affects individual knowledge and collective knowledge storage in the environment. A sudden decline in knowledge storage reduces collective or individual output and organizational productivity. According to the disruption cycle and the innovation organization learning cycle, 12 events segments, namely *t = 12*, specifically:

$$K_{c}\left(12\right)=K_{c}\left(0\right)+\left[1-\varphi_{c}\right]\left[1-\varphi_{c}\right]\left[K_{c}\left(12^{-}\right)-K_{c}\left(0\right)\right]$$ (1)

$$K_{i}\left(12\right)=K_{i}\left(0\right)+\left[1-\varphi_{i}\right]\left[K_{i}\left(12^{-}\right)-K_{i}\left(0\right)\right]$$ (2)

*t =* $12^{-}$ represents the time at the early stage12, $\;{\_}_{\text{c}}$ and ${\_}_{\text{i}}$ represent the disruption intensity of collectives and individuals, through $\;{\_}_{\text{c}}$ and ${\_}_{\text{i}}$ changing from 0.0 to 1.0, we can simulate any scenario from extreme organization stability to extreme disruption.

*Organization learning*. Disruption events have a particular impact on both individual and collective learning. Based on the mathematical equation described for the basic learning system and combining different variables on the learning and knowledge task completion rate in time *t*, we believe this function has an effect in both individual and collective learning:

$$q\left(t\right)=q_{0}c\left(t\right)i\left(t\right)=c\left(t\right)i\left(t\right)$$ (3)

Where c(t) represents the collective productivity and i(t) represents the average individual productivity, in the absence of general interference, we set $q_{0}=1$ . To simulate the impact of collective productivity, we utilized the organizational learning curve, often referred to as a *learning-by-doing*, which uses accumulation output representing knowledge acquisition through production and represents productivity as the necessary labor hours to produce a unit of product. We, therefore, define collective productivity as:

$$c\left(t\right)={\left[\frac{K_{c}\left(t\right)}{K_{c}\left(0\right)}\right]}^{\gamma_{c}}$$ (4)

$K_{c}\left(t\right)$ representing the collective knowledge storage in time t *,* $\gamma_{c}$ is the collective learning curve parameters, which creates the law of decreasing returns in the accumulation yield. In most of empirical studies, the range value $\gamma_{c}$ is [0,1]. In the case of accompanying knowledge accumulation with task completion and organizational forgetting, we simulated the forgetting rate of collective knowledge:

$$\frac{dK_{c}\left(t\right)}{dt}=q\left(t\right)-x_{c}K_{c}\left(t\right)$$ (5)

Collective knowledge $K_{c}\left(t\right)$ increases on the q(t) basis of unit time task completion rate. However, the description of our model also mentions that the forgetting phenomenon of knowledge decreases with time (3). This typical model represents the continuous depreciation process of collective knowledge savings and affects the collective yield. When $x_{c}$ increases, the total number of forgetting will become smaller. At the same time, we believe that individual learning is part of the basic learning systemg. The individual task completion rate similarly follows the learning curve and is described as the formation of collective knowledge. Although individual and collective learning curves are similar in formation, since individual and collective tend to accumulate different types of knowledge types at different types of rates, we define the individual learning curve as:

$$i\left(t\right)={\left[\frac{K_{i}\left(t\right)}{K_{i}\left(0\right)}\right]}^{\gamma_{i}}$$ (6)

And the individual knowledge forgetting rate:

$$\frac{dK_{i}\left(t\right)}{dt}=q\left(t\right)-x_{i}K_{i}\left(t\right)$$ (7)

Although individuals have different parameter values from the collective learning curves, they have the same threshold. At the same time, individual turnover and restructuring, task and technology change, and extreme events will influence the change of individual collective knowledge, learning rate, productivity, task completion rate, and forgetting rate and form a unified, systematic problem. Therefore, we use MATLAB 2016a to simulate all the variables included in the system systematically and derive the different influences of disruption events on organizational learning in the innovation cycle.

### Simulation Setup

The experimental simulation design in this paper analyzes the impact of disruption events on individual knowledge, collective knowledge, and knowledge acquisition while considering different stages of organizational innovation. This deep simulation confirms the influence of disruption events on the dual learning methods. Various degrees of disruption events effect individual learning and collective learning. In the second part, we change parameters ${\_}_{\text{c}}$ and ${\_}_{\text{i}}$ and test the extent of varying degrees of disruption events on organizational learning. At the same time, the effect of 100 simulation runs ${\_}_{\text{c}}$ and ${\_}_{\text{i}}$ on collective learning. Thus, with the average effect of the initial conditions of the organization and environment, the effect of the disruption event on organization learning shows on average over 10,000 simulation runs (100 observations in 100 different environments). Finally, we designed other unperformed model parameters initially randomly configured as 1,024 (2^10^), and these quantitative variable simulations completed stochastic significance detection of independent variables.

### Simulation Results

**FIGURE 3:**
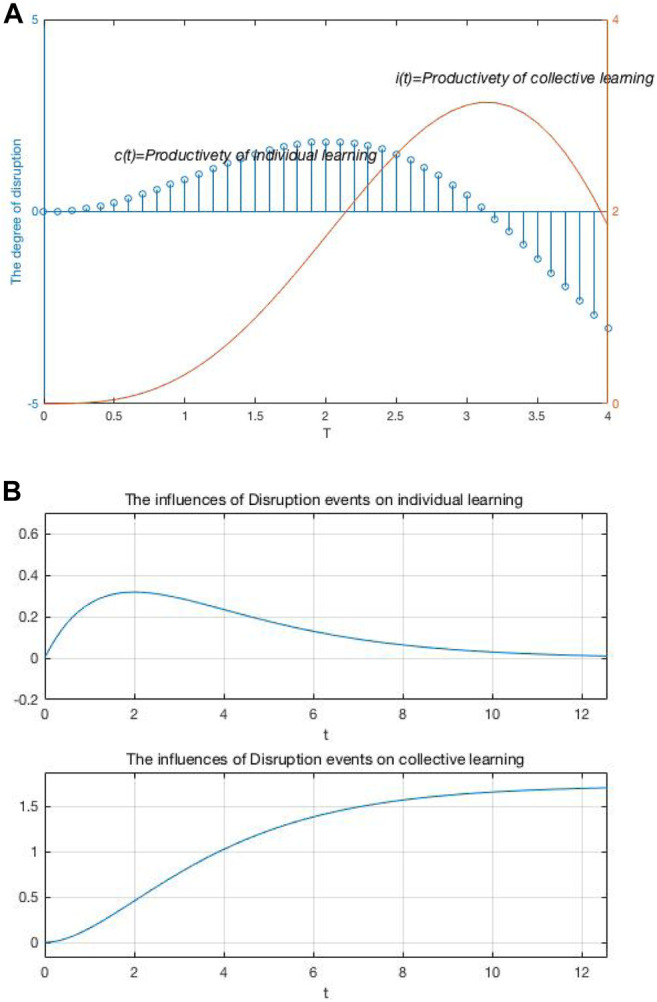
Influence of disruption on individual and collective learning.

Figure 3A shows changes in individual versus collective learning productivity under various degrees of disruption. Unlike existing conclusions, the simulation results found that individual learning productivity is extremely low when no disruption events occur inside and outside the organization. At the beginning of the disruption, the individual knowledge change, strong ties no longer exist. The disruption destroys the internal structure of the innovation network, and individuals enter the stage of rapid learning. Moreover, Figure 3B shows the changes in individual learning and collective learning under the influence of the same degree during the *t = 12* innovation cycle: individual learning rises rapidly during the innovation cycle, and collective learning rises steadily during the innovation cycle. This simulation result shows that in the long term, due to the negative impact of disruption events on individual knowledge, existing knowledge network, and decision path dependence, a large number of heterogeneous knowledge and resources flow into the organization. This inflow updates individual knowledge and has a significant effect on promoting collective learning in the long term.

## Discussion

How do exploration and exploitation affect OA in the context of inside and outside disruption events? As previously noted, the bulk of the literature on organizational learning emphasized that exploration and exploitation have significant importance for performance in the short and long terms. Considering the effects of inside and outside disruption is new to the field.

This study developed a dynamic environment variable for an organization’s inside (including restructuring, employee turnover, and task change) and outside (including changes in market structure, technology, and extreme events) exploration and exploitation, which is more specific and systematic than previous research. This study also set out to determine how DE moderates exploration and exploration. Our results show that, as predicted, the DE moderate the relationship between exploration and exploitation learning. DE has a complementary effect on OA. This finding equates to learning race and dynamic disruption arguments that proposed and other developments in that domain. However, more importantly, we do not find that the positive influence of exploitation learning—relative to exploration learning—on OA is stronger during ID events. Both exploration and exploitation have a significant and positive effect on OA during ID. However, our empirical research results show that the positive effect of exploration learning on OA is stronger during ID than is the effect of exploitation. Two reasons likely exist: First, exploitation learning is beneficial for OA. Exploitation learning not only needs tacit knowledge but also depends on the organization’s strong ties. However, ID, including restructuring, employee turnover, and task change, brings more heterogeneous explicit knowledge and weak ties, which are bad for exploitation. Second, exploration learning improves long-term recoverability, which benefits organizational adaptation during ID. The findings support hypothesis 3 and demonstrate that, during outside disruption events, exploration has a stronger positive effect on OA than exploitation. Surprisingly, prior literature finds that both exploitation and exploration are beneficial. Our results show that during outside disruption, exploitation learning has significant and negative effects on OA. This result suggests that the firm takes advantage of different learning methods, increasing OA and surviving DE.

### Theoretical Contribution

First, this study contributes to innovation theory and highlights the role of disruption events (DE). We found that inside and outside disruption do not harm but instead benefit OA. Our research pioneers conceptualizing, operationalizing, and measuring DE and its effect on OA. We divide the DE into two parts: ID events, including restructuring, employee turnover, and task change, which take place within the organizational environment. The other part is outside disruption events, including changes in market structure, changes in technology, and extreme events that occur outside the organization. We highlight the different effects of exploration and exploitation on OA during inside and outside disruptions. This disparate influence is essential for exploration and exploitation but has received little attention in the literature.

Second, this study opens a new avenue of inquiry that offers considerable promise in explaining organizational adaptation (OA) to gain better OA. Furthermore, investigating other strategies for firm learning to improve performance under DE may be promising. In essence, our results suggest important interactions among exploration, exploitation, and OA during ID and OD events.

Finally, our study also has implications for the burgeoning stream of research on exploration and exploitation. Prior literature always suggested that exploration and exploitation benefit organizational performance. The distinction between exploration and exploitation learning highlights the long- and short-term performance and different ties and knowledge that individuals gain from the learning. However, our research argued that exploration and exploitation learning might be negative for OA under different circumstances. For example, strong ties and tacit knowledge will be useless or obsolete when technology or tasks change. Employee turnover hurts organizational performance and disrupts existing knowledge and routines for interacting and accomplishing tasks. Thus, our study provides new insights into understanding exploration and exploitation for OA.

### Practical Implications

This study also contributes to the research on practices of organizational governance and policy implications. First, the results suggest that as a shift variable disruption events do not always harm organizational adaptation. So firms need to make some policies to nurture a shared vision to promote revolution or design incentive mechanisms to enhance diversity resources. Second, the results indicate that exploitation is more beneficial for organizational adaptation during inside disruption, and exploration is more effective during outside disruption. Accordingly, firms can benefit from exploitation and exploration to gain a competitive advantage to realize sustainable development. Third, this study finds that exploitation does not always benefit the organizational adaptation during the different kinds of disruption events. Firms should avoid concentrating resources on exploitation during outside disruption.

### Limitations and Directions for Future Research

This study contains limitations relevant to consider when interpreting the reported results. First, we proxied DE using two dimensions—inside and outside disruption events. Although we believe that these dimensions are reasonable proxies in our context, the fact of the matter is that disruption is rather dynamic. The analysis excludes the dynamic nature of disruption processes that affect an organization’s learning and OA. Additionally, this study examines a unitary industry using our data from Alibaba, Meituan, Dianping, Baidu, Beibei, TP-Link, and Maxio that only represent the information technology and Internet company context. The research in this paper excludes other industry disruption actors, such as productivity change, that likely would play a key role in other empirical contexts. Third, the effect of two learning methods on OA under DE not only affects the organizational level. Our study’s analytical approach allows us to examine and test for relationships consistent with static processes. However, it is important to remember that we cannot directly test for causal relationships in our data.

Future research can untangle cross-level learning relationships among organizations and individual-level actors, focusing on the influence of disruption on the evolution of organizational learning.

## Data Availability

The original contributions presented in the study are included in the article/Supplementary Material, further inquiries can be directed to the corresponding author.

## Footnotes

1 As Granovetter (1973) mentioned in *The Strength of Weak Ties*, measuring ties could use four dimensions: a combination of the amount of time, the emotional intensity, the intimacy, and the reciprocal services. We understand that turnover is a type of disruptive event that affects organizational adaptation, but measuring the sub-dimension of employee turnover is not needed

## References

[B1] Aggarwal V. A. Siggelkow N. Singh H. (2011). Governing Collaborative Activity: Interdependence and the Impact of Coordination and Exploration. Strat. Mgmt. J. 32 (7), 705–730. 10.1002/smj.900

[B2] Ali Hameed A. Karlik B. Salman M. S. Eleyan G. (2019). Robust Adaptive Learning Approach to Self-Organizing Maps. Knowledge-Based Syst. 171, 25–36. 10.1016/j.knosys.2019.01.011

[B3] Amburgey T. L. Kelly D. Barnett W. P. (1993). Resetting the Clock: The Dynamics of Organizational Change and Failure. Administrative Sci. Q. 38 (1), 51–73. 10.2307/2393254

[B4] Anderson E. G. Jr Lewis K. (2014). A Dynamic Model of Individual and Collective Learning amid Disruption. Organ. Sci. 25 (2), 356–376. 10.1287/orsc.2013.0854

[B5] Angeler D. G. Fried-Petersen H. B. Allen C. R. Garmestani A. Twidwell D. Chuang W.-C. (2019). “Adaptive Capacity in Ecosystems,”in. Advances in Ecological Research. Editors Bohan D. A. Dumbrell A. J. (Academic Press), 60, 1–24. 10.1016/bs.aecr.2019.02.001 PMC6944309 31908359

[B6] Aranda C. Arellano J. Davila A. (2017). Organizational Learning in Target Setting. Amj 60 (3), 1189–1211. 10.5465/amj.2014.0897

[B7] Barnett W. P. Freeman J. (2001). Too Much of a Good Thing? Product Proliferation and Organizational Failure. Organ. Sci. 12 (5), 539–558. 10.1287/orsc.12.5.539.10095

[B8] Bode C. Wagner S. M. Petersen K. J. Ellram L. M. (2011). Understanding Responses to Supply Chain Disruptions: Insights from Information Processing and Resource Dependence Perspectives. Amj 54 (4), 833–856. 10.5465/AMJ.2011.64870145

[B9] Boumgarden P. Nickerson J. Zenger T. R. (2012). Sailing into the Wind: Exploring the Relationships Among Ambidexterity, Vacillation, and Organizational Performance. Strat. Mgmt. J. 33 (6), 587–610. 10.1002/smj.1972

[B11] Carnabuci G. (2010). The Ecology of Technological Progress: How Symbiosis and Competition Affect the Growth of Technology Domains. Social Forces 88, 2163–2187. 10.1353/sof.2010.0045

[B12] Choi S. McNamara G. (2018). Repeating a Familiar Pattern in a New Way: T He Effect of Exploitation and Exploration on Knowledge Leverage Behaviors in Technology Acquisitions. Strat Mgmt J. 39 (2), 356–378. 10.1002/smj.2677

[B13] Christensen C. M. Bower J. L. (1996). Customer Power, Strategic Investment, and the Failure of Leading Firms. Strat. Mgmt. J. 17 (3), 197–218. 10.1002/(sici)1097-0266(199603)17:3<197:aid-smj804>3.0.co;2-u

[B14] Cohendet P. S. Simon L. O. (2016). Always Playable: Recombining Routines for Creative Efficiency at Ubisoft Montreal's Video Game Studio. Organ. Sci. 27 (3), 614–632. 10.1287/orsc.2016.1062

[B15] Cui V. Yang H. Vertinsky I. (2018). Attacking Your Partners: Strategic Alliances and Competition between Partners in Product Markets. Strat Mgmt J. 39 (12), 3116–3139. 10.1002/smj.2746

[B16] Dattée B. Alexy O. Autio E. (2018). Maneuvering in Poor Visibility: How Firms Play the Ecosystem Game when Uncertainty Is High. Amj 61 (2), 466–498. 10.5465/amj.2015.0869

[B17] Desai V. (2015). Learning through the Distribution of Failures within an Organization: Evidence from Heart Bypass Surgery Performance. Amj 58 (4), 1032–1050. 10.5465/amj.2013.0949

[B18] Døjbak Håkonsson D. Eskildsen J. K. Argote L. Mønster D. Burton R. M. Obel B. (2016). Exploration versus Exploitation: Emotions and Performance as Antecedents and Consequences of Team Decisions. Strat. Mgmt. J. 37 (6), 985–1001. 10.1002/smj.2380

[B19] Donald H. William S. A. (2016). Two-phase Longitudinal Model of a Turnover Event:disruption,recovery Rates,and Moderators of Collective Performance. Acad. Manage. J. 59 (3), 906–929. 10.5465/amj.2013.0546

[B20] Dowell G. W. S. Muthulingam S. (2017). Will Firms Go green if it Pays? T He Impact of Disruption, Cost, and External Factors on the Adoption of Environmental Initiatives. Strat. Mgmt. J. 38 (6), 1287–1304. 10.1002/smj.2603

[B22] Engman A. (2019). Embodiment and the Foundation of Biographical Disruption. Soc. Sci. Med. 225, 120–127. 10.1016/j.socscimed.2019.02.019 30825759

[B23] Gambeta E. Koka B. R. Hoskisson R. E. (2019). Being Too Good for Your Own Good: A Stakeholder Perspective on the Differential Effect of Firm‐employee Relationships on Innovation Search. Strat Mgmt J. 40 (1), 108–126. 10.1002/smj.2967

[B24] Gimeno J. Woo C. Y. (1996). Hypercompetition in a Multimarket Environment: The Role of Strategic Similarity and Multimarket Contact in Competitive De-escalation. Organ. Sci. 7 (3), 322–341. 10.1287/orsc.7.3.322

[B25] Granovetter M. S. (1973). The Strength of Weak Tie. Am. J. Sociol. 42 (3), 1360–1380. 10.1086/225469

[B26] Haans R. F. J. (2019). What's the Value of Being Different when Everyone Is? the Effects of Distinctiveness on Performance in Homogeneous versus Heterogeneous Categories. Strat Mgmt J. 40 (1), 3–27. 10.1002/smj.2978

[B27] Hale D. Jr Ployhart R. E. Shepherd W. (2016). A Two-phase Longitudinal Model of A Turnover Event: Disruption, Recovery Rates, and Moderators of Collective PerformancE. Amj 59 (3), 906–929. 10.5465/amj.2013.0546

[B28] Hoever I. J. Zhou J. van Knippenberg D. (2018). Different Strokes for Different Teams: The Contingent Effects of Positive and Negative Feedback on the Creativity of Informationally Homogeneous and Diverse Teams. Amj 61 (6), 2159–2181. 10.5465/amj.2016.0642

[B29] Ioannou I. (2014). When Do Spinouts Enhance Parent Firm Performance? Evidence from the U.S. Automobile Industry, 1890-1986. Organ. Sci. 25 (2), 529–551. 10.1287/orsc.2013.0846

[B30] Jain A. (2016). Learning by Hiring and Change to Organizational Knowledge: Countering Obsolescence as Organizations Age. Strat. Mgmt. J. 37 (8), 1667–1687. 10.1002/smj.2411

[B31] Jarzabkowski P. A. Lê J. K. (2012). Toward a Theory of Coordinating: Creating Coordinating Mechanisms in Practice. Organ. Sci. 23 (4), 907–927. 10.1287/orsc.1110.0693

[B32] Jiang H. Cannella A. A. Xia J. Semadeni M. (2017). Choose to Fight or Choose to Flee? A Network Embeddedness Perspective of Executive Ship Jumping in Declining Firms. Strat. Mgmt. J. 38 (10), 2061–2079. 10.1002/smj.2637

[B33] Kaiser U. Kongsted H. C. Laursen K. Ejsing A.-K. (2018). Experience Matters: The Role of Academic Scientist Mobility for Industrial Innovation. Strat. Mgmt. J. 39 (7), 1935–1958. 10.1002/smj.2907

[B34] Kilkki K. Mäntylä M. Karhu K. Hämmäinen H. Ailisto H. (2018). A Disruption Framework. Technol. Forecast. Soc. Change 129, 275–284. 10.1016/j.techfore.2017.09.034

[B35] Kim C. Y. Lim M. S. Yoo J. W. (2019). Ambidexterity in External Knowledge Search Strategies and Innovation Performance: Mediating Role of Balanced Innovation and Moderating Role of Absorptive Capacity. Sustainability 11 (18), 5111. 10.3390/su11185111

[B36] Kim S. Anand J. J. (2018). Knowledge Complexity and the Performance of Inter-unit Knowledge Replication Structures. Strat. Mgmt. J. 39 (7), 1959–1989. 10.1002/smj.2899

[B38] Laureiro-Martínez D. Brusoni S. Canessa N. Zollo M. (2015). Understanding the Exploration-Exploitation Dilemma: An fMRI Study of Attention Control and Decision-Making Performance. Strat. Mgmt. J. 36 (3), 319–338. 10.1002/smj.2221

[B39] Madsen P. M. Desai V. (2010). Failing to Learn? the Effects of Failure and Success on Organizational Learning in the Global Orbital Launch Vehicle Industry. Amj 53 (3), 451–476. 10.5465/AMJ.2010.51467631

[B40] March J. G. (19911991). Exploration and Exploitation in Organizational Learning. Organ. Sci. 2 (1), 71–87. 10.1287/orsc.2.1.71

[B41] Mata J. Alves C. (2018). The Survival of Firms Founded by Immigrants: Institutional Distance between home and Host Country, and Experience in the Host Country. Strat. Mgmt. J. 39 (11), 2965–2991. 10.1002/smj.2945

[B42] Mevada J. Devi S. Pandit A. (2019). Large Scale Microbial Cell Disruption Using Hydrodynamic Cavitation: Energy Saving Options. Biochem. Eng. J. 143, 151–160. 10.1016/j.bej.2018.12.010

[B43] Moreira S. Markus A. Laursen K. (2018). Knowledge Diversity and Coordination: The Effect of Intrafirm Inventor Task Networks on Absorption Speed. Strat Mgmt J. 39 (9), 2517–2546. 10.1002/smj.2914

[B44] Mudambi R. Swift T. (2014). Knowing when to Leap: Transitioning between Exploitative and Explorative R&D. Strat. Mgmt. J. 35 (1), 126–145. 10.1002/smj.2097

[B45] Park J. Park C. Gye M. C. Lee Y. (2019). Assessment of Endocrine-Disrupting Activities of Alternative Chemicals for Bis(2-Ethylhexyl)phthalate. Environ. Res. 172, 10–17. 10.1016/j.envres.2019.02.001 30769184

[B46] Paruchuri S. Awate S. (2017). Organizational Knowledge Networks and Local Search: The Role of Intra‐organizational Inventor Networks. Strat. Mgmt. J. 38 (3), 657–675. 10.1002/smj.2516

[B47] Paruchuri S. Nerkar A. Hambrick D. C. (2006). Acquisition Integration and Productivity Losses in the Technical Core: Disruption of Inventors in Acquired Companies. Organ. Sci. 17 (5), 545–562. 10.1287/orsc.1060.0207

[B48] Piao M. Zajac E. J. (2016). How Exploitation Impedes and Impels Exploration: Theory and Evidence. Strat. Mgmt. J. 37 (7), 1431–1447. 10.1002/smj.2402

[B49] Posen H. E. Leiblein M. J. Chen J. S. (2018). Toward a Behavioral Theory of Real Options: Noisy Signals, Bias, and Learning. Strat Mgmt J. 39 (4), 1112–1138. 10.1002/smj.2757

[B50] Ren B. Zhang Y. Chen J. Shen L. (2019). Efficient Network Disruption under Imperfect Information: The Sharpening Effect of Network Reconstruction with No Prior Knowledge. Physica A: Stat. Mech. its Appl. 520, 196–207. 10.1016/j.physa.2018.12.009

[B51] Rhee L. Leonardi P. M. (2018). Which Pathway to Good Ideas? A N Attention‐based View of Innovation in Social Networks. Strat Mgmt J. 39 (4), 1188–1215. 10.1002/smj.2755

[B52] Sawik T. (2019). Disruption Mitigation and Recovery in Supply Chains Using Portfolio Approach. Omega 84, 232–248. 10.1016/j.omega.2018.05.006

[B53] Schilling M. A. Fang C. (2014). When Hubs Forget, Lie, and Play Favorites: Interpersonal Network Structure, Information Distortion, and Organizational Learning. Strat. Mgmt. J. 35 (7), 974–994. 10.1002/smj.2142

[B54] Schumpeter J. A. (1939). Business Cycles: A Theoretical, Historical, and Statistical Analysis of the Capitalist Process. New York, NY: McGraw-Hill Book Company, Inc.

[B55] Sørensen K. T. (2019). Asasim: Adaptive Sampling for Electromagnetic Simulations. Comput. Phys. Commun. 236, 268–273. 10.1016/j.cpc.2018.10.017

[B56] Stettner U. Lavie D. (2014). Ambidexterity under Scrutiny: Exploration and Exploitation via Internal Organization, Alliances, and Acquisitions. Strat. Mgmt. J. 35 (13), 1903–1929. 10.1002/smj.2195

[B57] Stieglitz N. Knudsen T. Becker M. C. (2016). Adaptation and Inertia in Dynamic Environments. Strat. Mgmt. J. 37 (9), 1854–1864. 10.1002/smj.2433

[B58] Swift T. (2016). The Perilous Leap between Exploration and Exploitation. Strat. Mgmt. J. 37 (8), 1688–1698. 10.1002/smj.2423

[B59] Uzunca B. (2018). A Competence-Based View of Industry Evolution: The Impact of Submarket Convergence on Incumbent−Entrant Dynamics. Amj 61 (2), 738–768. 10.5465/amj.2015.1080

[B60] Vega J. Hernández F. Dormido-Canto S. Isayama A. Joffrin E. Matsunaga G. (2019). Assessment of Linear Disruption Predictors Using JT-60U Data. Fusion Eng. Des. 146, 1291–1294. 10.1016/j.fusengdes.2019.02.061

[B61] Yan J. Z. Chang S. J. (2018). The Contingent Effects of Political Strategies on Firm Performance: A Political Network Perspective. Strat Mgmt J. 39 (8), 2152–2177. 10.1002/smj.2908

[B62] Yang H. Phelps C. Steensma H. K. (2010). Learning from what Others Have Learned from You: The Effects of Knowledge Spillovers on Originating Firms. Amj 53 (2), 371–389. 10.5465/AMJ.2010.49389018

[B63] Yang H. Zheng Y. Zaheer A. (2015). Asymmetric Learning Capabilities and Stock Market Returns. Amj 58 (2), 356–374. 10.5465/amj.2012.0675

[B64] Yang H. Zheng Y. Zhao X. (2014). Exploration or Exploitation? Small Firms' alliance Strategies with Large Firms. Strat. Mgmt. J. 35 (1), 146–157. 10.1002/smj.2082

[B65] Yu W. Minniti M. Nason R. (2019). Underperformance Duration and Innovative Search: Evidence from the High‐tech Manufacturing Industry. Strat Mgmt J. 40 (5), 836–861. 10.1002/smj.2988

