# Supplementary material for: THE Impact of Disruption on the Relationship Between Exploitation, Exploration, and Organizational Adaptation
Source: Front Sociol. 2021 Nov 3;6:757160. doi: 10.3389/fsoc.2021.757160 (PMC8595394; doi:10.3389/fsoc.2021.757160)
Supplement: Supplementary file 1 [file datasheet1.zip › 757160_soberg_Appendix_A.docx]

**Appendix A.**

Robustness test 1:

| VARIABLE | IA+CA | | | | | | | | |
| --- | --- | --- | --- | --- | --- | --- | --- | --- | --- |
| Control variables | M1 | M2 | M3 | | | M4 | | M5 |  |
| Firm age | 0.089* | 0.102* | 0.211* | 0.263* | 0.176* | 0.152* | 0.209* | |  |
| Firm size | 0.192 | 0.262 | 0.152 | 0.267 | 0.128 | 0.163 | 0.103 | |  |
| Knowledge  diversity | 0.196* | 0.233* | 0.128* | 0.149* | 0.161* | 0.205* | 0.314* | |  |
| Accumulated experience diversity | 0.152* | 0.163* | 0.141* | 0.146* | 0.159* | 0.209* | 0.139* | |  |
| Technological dynamism | 0.158* | 0.218* | 0.209* | 0.189* | 0.177* | 0263* | 0.182* | |  |
| Independent variable |  |  |  |  |  |  |  | |  |
| ER |  | 0.209* | 0.136* | 0.162* | 0.228* | 0.112* | 0.204* | |  |
| ET |  | 0.129** | 0.266* | 0.212** | 0.231* | 0.249** | 0.251** | |  |
| ID |  | -0.324* | 0.259 | 0.301 | 0.211 | 0.267 | 0.145 | |  |
| OD |  | -0.146 | 0.178 | -0.118 | -0.302 | -0.189* | 0.112* | |  |
| ID*EA |  |  | 0.254** |  | 0.163* |  |  | |  |
| ID*EI |  |  |  | 0.214** | 0.128* |  |  | |  |
| OD*EA |  |  |  |  |  | 0.246** |  | |  |
| OD*EI |  |  |  |  |  | -0.131** |  | |  |
| DE*EAL*EI |  |  |  |  |  |  | 0.225** | |  |
| Observations | 132 | 132 | 132 | 132 | 132 | 132 | 132 | |  |
| R^2^ | 0.079 | 0.102 | 0.196 | 0.184 | 0.209 | 0.236 | 0.257 | |  |
| Adjusted R | 0.046 | 0.185 | 0.193 | 0.180 | 0.206 | 0.234 | 0.254 | |  |
| F | 4.332* | 4.258** | 4.122** | 4.162* | 4.155* | 4.189* | 3.859** | |  |
