# Supplementary material for: THE Impact of Disruption on the Relationship Between Exploitation, Exploration, and Organizational Adaptation
Source: Front Sociol. 2021 Nov 3;6:757160. doi: 10.3389/fsoc.2021.757160 (PMC8595394; doi:10.3389/fsoc.2021.757160)
Supplement: Supplementary file 1 [file datasheet1.zip › 757160_soberg_Appendix_B.docx]

**Appendix B.**

Robustness test 2:

| VARIABLE | OA | | | | | | | | |
| --- | --- | --- | --- | --- | --- | --- | --- | --- | --- |
| Control variables | M1 | M2 | M3 | | | M4 | | M5 |  |
| Firm age | 0.139* | 0.157* | 0.166* | 0.211* | 0.189* | 0.174* | 0.209* | |  |
| Firm size | 0.184 | 0.209 | 0.153 | 0.261 | 0.142 | 0.171 | 0.122 | |  |
| Knowledge  diversity | 0.125* | 0.214* | 0.112* | 0.186* | 0.126* | 0.166* | 0.200* | |  |
| Accumulated experience diversity | 0.200* | 0.250* | 0.214* | 0.129* | 0.164* | 0.211* | 0.141* | |  |
| technological capability and financing capability | 0.109* | 0.332* | 0.320* | 0.247* | 0.211* | 0.255* | 0.269* | |  |
| Independent variable |  |  |  |  |  |  |  | |  |
| ER |  | 0.189* | 0.167* | 0.177* | 0.187* | 0.200* | 0.211* | |  |
| ET |  | 0.162** | 0.188* | 0.183** | 0.199* | 0.164** | 0.209** | |  |
| ID |  | -0.205 | 0.230 | 0.306 | 0.0169 | 0.020* | 0.184* | |  |
| OD |  | -0.149 | 0.176 | -0.125 | -0.303 | -0.218 | 0.211 | |  |
| ID*EA |  |  | 0.184** |  | 0.228* |  |  | |  |
| ID*EI |  |  |  | 0.214** | 0.210* |  |  | |  |
| OD*EA |  |  |  |  |  | 0.196** |  | |  |
| OD*EI |  |  |  |  |  | -0.132** |  | |  |
| DE*EAL*EI |  |  |  |  |  |  | 0.266** | |  |
| Observations | 132 | 132 | 132 | 132 | 132 | 132 | 132 | |  |
| R^2^ | 0.056 | 0.109 | 0.189 | 0.204 | 0.236 | 0.257 | 0.296 | |  |
| Adjusted R | 0.053 | 0.107 | 0.188 | 0.203 | 0.231 | 0.251 | 0.293 | |  |
| F | 3.158* | 3.274** | 3.699** | 3.366* | 4.096* | 4.151* | 4.020* | |  |
