# Supplementary material for: THE Impact of Disruption on the Relationship Between Exploitation, Exploration, and Organizational Adaptation
Source: Front Sociol. 2021 Nov 3;6:757160. doi: 10.3389/fsoc.2021.757160 (PMC8595394; doi:10.3389/fsoc.2021.757160)
Supplement: Supplementary file 1 [file datasheet1.zip › 757160_soberg_Appendix_C.docx]

**Appendix C.**

Robustness test 3:

| VARIABLE | OIP | | | | | | | | |
| --- | --- | --- | --- | --- | --- | --- | --- | --- | --- |
| Control variables | M1 | M2 | M3 | | | M4 | | M5 |  |
| Firm age | 0.156* | 0.218* | 0.165* | 0.215* | 0.155* | 0.126* | 0.213* | |  |
| Firm size | 0.104* | 0.221 | 0.136* | 0.206 | 0.188 | 0.112 | 0.190 | |  |
| Knowledge  diversity | 0.098* | 0.125* | 0.109* | 0.178* | 0.135* | 0.164* | 0.126* | |  |
| Accumulated experience diversity | -0.248 | -0.169 | 0.174 | -0.135 | 0.150 | -0.200 | 0.125 | |  |
| Technological dynamism | 0.136* | 0.279* | 0.233* | 0.124* | 0.111* | 0276* | 0.245* | |  |
| Independent variable |  |  |  |  |  |  |  | |  |
| ER |  | 0.178* | 0.123* | 0.102* | 0.264* | 0.109* | 0.177* | |  |
| ET |  | 0.116** | 0.268* | 0.239** | 0.271* | 0.209** | 0.244** | |  |
| ID |  | -0.203 | 0.322 | 0.320 | 0.021 | 0.314* | 0.154* | |  |
| OD |  | -0.222 | 0.280 | -0.299 | -0.231 | -0.411 | 0.149* | |  |
| ID*EA |  |  | 0.107** |  | 0.132* |  |  | |  |
| ID*EI |  |  |  | 0.201** | 0.179* |  |  | |  |
| OD*EA |  |  |  |  |  | 0.120** |  | |  |
| OD*EI |  |  |  |  |  | -0.183** |  | |  |
| DE*EAL*EI |  |  |  |  |  |  | 0.227** | |  |
| Observations | 132 | 132 | 132 | 132 | 132 | 132 | 132 | |  |
| R^2^ | 0.068 | 0.174 | 0.221 | 0.215 | 0.239 | 0.249 | 0.258 | |  |
| Adjusted R | 0.066 | 0.173 | 0.220 | 0.213 | 0.238 | 0.247 | 0.257 | |  |
| F | 3.222* | 4.188** | 3.668** | 3.221* | 4.198* | 3.226* | 3.245** | |  |

OIP: Organization Innovation Performance.
